# Focal point-by-point pulsed field ablation for the treatment of atrial arrhythmias in patients with challenging anatomy where radiofrequency ablation cannot be applied: A case series

**DOI:** 10.1016/j.hrcr.2023.10.012

**Published:** 2023-10-14

**Authors:** Florent Farnir, Justin Luermans, Randolph Manusama, Dennis den Uijl, Sevasti Maria Chaldoupi, Dominik Linz

**Affiliations:** ∗ Department of Cardiology, Maastricht University Medical Centre and Cardiovascular Research Institute Maastricht, Maastricht, The Netherlands; † Department of Cardiology, Zuyderland Medical Centre, Heerlen, The Netherlands; ‡ Department of Biomedical Sciences, Faculty of Health and Medical Sciences, University of Copenhagen, Copenhagen, Denmark

**Keywords:** Pulsed field ablation, Focal ablation, High-risk ablation, Electrophysiology, Safety

## Introduction

**Key Teaching Points**

• Focal point-by-point pulsed field ablation (F-PFA) should be considered as an alternative to thermal ablation in patients with challenging anatomies.
• F-PFA systems allow the creation of tailored ablation lesion sets for complex and targeted ablation procedures.
• The safety and achievement of intraprocedural endpoints and short-term outcomes in patients with challenging anatomies are promising.

Catheter ablation is the cornerstone treatment of atrial arrhythmias by restoring sinus rhythm, reducing symptoms, and improving quality of life.^1^^,^^2^ Thermal energy sources, including radiofrequency energy and cryoenergy, have been well studied and demonstrated to be effective.^3^ However, they are associated with a risk of damage of the adjacent organs and anatomical structures owing to thermal conduction. Secondary damage to the phrenic nerve, esophagus (including a hiatal hernia), and surrounding vessels is of particular concern owing to proximity to the heart.^4^ Pulsed field ablation (PFA) is a novel nonthermal ablation technology. It consists of a high-voltage train with very short pulses, which results in tissue-specific damage in the heart by cardiomyocyte-selective electroporation.^5^ Recent studies, mainly using single-shot PFA devices, have demonstrated safety and efficacy in the treatment of atrial fibrillation (AF), without the risk of thermal damage of the neighboring tissues.^5^^,^^6^ In addition, focal point-by-point PFA (F-PFA) ablation systems have been introduced allowing the creation of lesion sets tailored to the patient’s anatomy.^7^ Among them, the CENTAURI (Galvanize EP, San Carlos, CA) system enables biphasic, monopolar F-PFA to be applied through commercially available contact force–sensing, solid-tip focal ablation catheters and visualized in the 3D electroanatomical mapping system (except for transient disappearance of the catheter during PFA delivery). PFA energy is delivered using a current of 22 A (7 pulse trains) for ablation of the posterior wall (ie, posterior portions of the pulmonary veins [PVs], posterior line of the posterior box) and a current of 25 A (10 pulse trains) for ablation of the anterior wall (anterior portions of the PVs, anterior lines, roof line of the posterior box). For ablation in the right atrium, a current of 22 A is used. Applications are guided by ablation tags of 6 mm placed with 20%–30% overlap (inter-lesion distance of 4 mm), using a contact force between 10 and 25 g and an irrigation rate of 4 mL/min.

In this case series we present 3 patients who were referred to our hospital for catheter ablation of symptomatic supraventricular tachycardia. All of them were initially declined because treatment by thermal radiofrequency ablation or cryoablation was deemed too risky owing to anatomic obstacles. Herein, we describe how we successfully used F-PFA to apply lesion sets tailored to the challenging anatomies without complications during the procedures and follow-up.

All patients gave written informed consent.

## Case report

### First case

A 64-year-old woman was referred to our hospital for a catheter ablation of symptomatic paroxysmal AF (EHRA score IIb). Further medical history included obesity (body mass index [BMI] 30 kg/m^2^), diabetes mellitus, ischemic heart disease (left ventricular ejection fraction of 39%), coronary artery bypass graft, and implantable cardioverter-defibrillator implantation for secondary prevention of ischemic ventricular tachycardia. The patient also underwent endovascular aorta repair in the context of an infrarenal aortic aneurysm 7 years ago.

**Figure 1:**
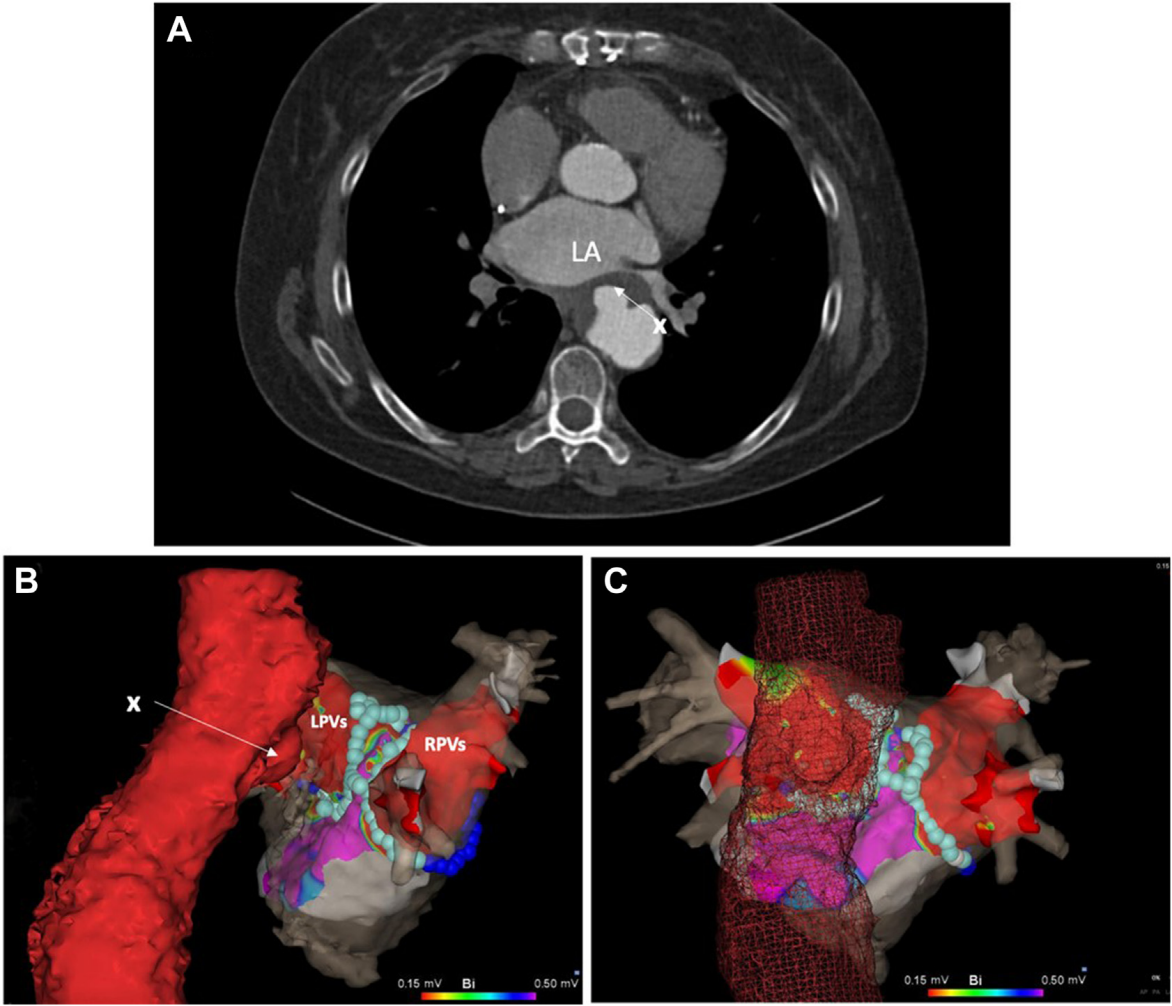
**A:** Cardiac computed tomography (CT) angiogram showing proximity between the saccular anterior aortic aneurysm and the left inferior pulmonary vein. **B, C:** Three-dimensional anatomical reconstruction integrating chest CT and 3-dimensional electroanatomical map (CARTO; Biosense Webster) demonstrating the proximity of the aneurysm to the applications of focal point-by-point pulsed field ablation (light blue balls) on the posterior wall of the left pulmonary veins (LPVs); right lateral (**B**) and posterior (**C**) views. LA = left atrium; RPVs = right pulmonary veins.

Routine preprocedural work-up included a cardiac computed tomography angiogram, which demonstrated a saccular aneurysm of the anterior portion of the descending aorta, intimately involved with the posterior wall of the left atrium (LA) and the posteroinferior part of the left inferior PV, with significant narrowing of its ostium (Figure 1A). After discussion during a multidisciplinary meeting, ablation by a thermal technology was deemed too risky, and the patient was scheduled for pulmonary vein isolation (PVI) by F-PFA.

After transfemoral access, a decapolar catheter was placed in the coronary sinus (Webster CS catheter; Biosense Webster, Inc, Diamond Bar, CA). Transesophageal echo-guided double transseptal access was performed and a high-density mapping catheter (PentaRay catheter; Biosense Webster) was used to perform electroanatomical reconstruction of the LA (CARTO; Biosense Webster). The ablation catheter (ThermoCool SmartTouch Catheter, Biosense Webster) was then advanced into the LA. PVI was performed as follows: The focal PFA generator (CENTAURI; Galvanize EP, San Carlos, CA) was connected to the CARTO system as described previously. Ipsilateral pair-wise wide antral isolation of the PVs was performed using F-PFA. Effective electrical first path isolation of the PVs was demonstrated. Anatomical reconstruction demonstrated a significant application of PFA energy opposite to the aortic aneurysm (Figure 1B–1D). No complications were noted during the procedure or in its immediate aftermath. An interrogation of the ICD revealed excellent lead and device parameters. There was no AF recurrence at 6 months.

### Second case

**Figure 2:**
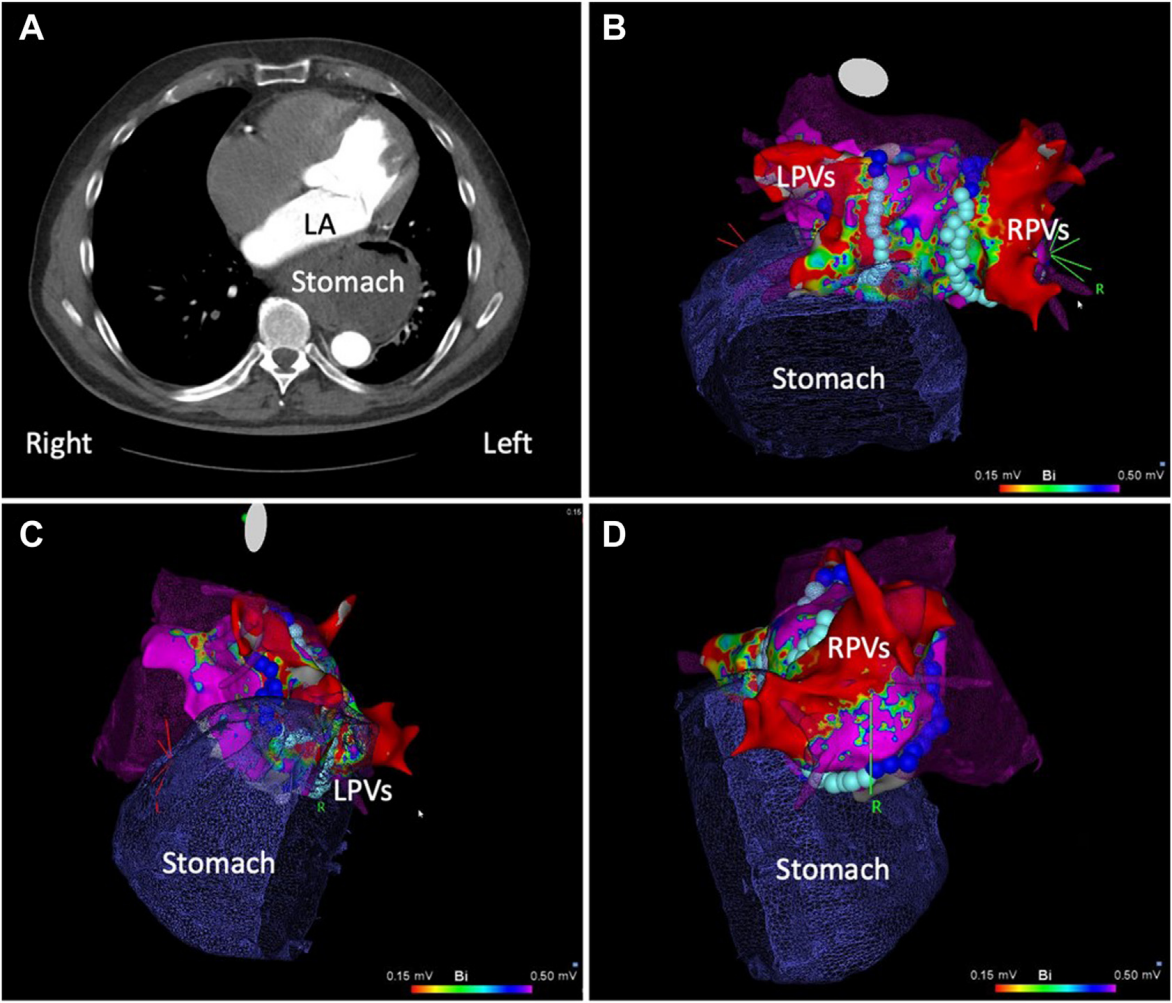
**A:** Cardiac computed tomography (CT) angiogram showing proximity between the herniated stomach and the left atrium (LA). **B–D:** Three-dimensional anatomical reconstruction integrating chest CT and 3-dimensional electroanatomical map (CARTO; Biosense Webster) demonstrating the proximity of the herniated stomach (shadowed dark blue) to the applications of focal point-by-point pulsed field ablation (light blue balls) on the posterior wall: **B**, posterior; **C**, left lateral; **D**, right lateral views. LPVs = left pulmonary veins; RPVs = right pulmonary veins.

A 63-year-old man was referred to our hospital for catheter ablation of symptomatic paroxysmal AF. Medical history included sleep apnea, overweight (BMI 26 kg/m^2^), and AF-related tachycardiomyopathy with a reduced systolic left ventricular function of 38%. The patient was treated with apixaban 5 mg twice a day and metoprolol 100 mg once a day. Preprocedural cardiac computed tomography angiogram revealed a hiatal hernia with herniation of part of the stomach into the thoracic cavity (Figure 2A). Because of the close proximity of the predicted LA ablation lines and the expected high risk of thermal damage to the herniated stomach during thermal radiofrequency ablation or cryoablation, the patient was planned for F-PFA ablation.

The same procedural setup as for the first case was used. The ablation was performed during AF, owing to early recurrence of AF after electrical cardioversion. Ipsilateral pair-wise wide antral isolation of the PVs was performed as described above for the first case. There was no gastric symptomatology in the immediate aftermath of the procedure and during follow-up. Magnetic resonance imaging (MRI) performed 3 months after the procedure showed no residual gastric lesions.

### Third case

A 39-year-old woman, BMI 31 kg/m^2^, with no previous cardiac history presented to the emergency department for palpitations and chest pain. The 12-lead ECG showed a regular narrow complex tachycardia at 208 beats per minute, with long RP and incomplete right bundle branch block. The tachycardia resolved on verapamil 10 mg (intravenous), after failure of adenosine 12 mg. The patient was then treated with verapamil 120 mg once a day. The patient was further referred to our center for electrophysiological study and ablation.

**Figure 3:**
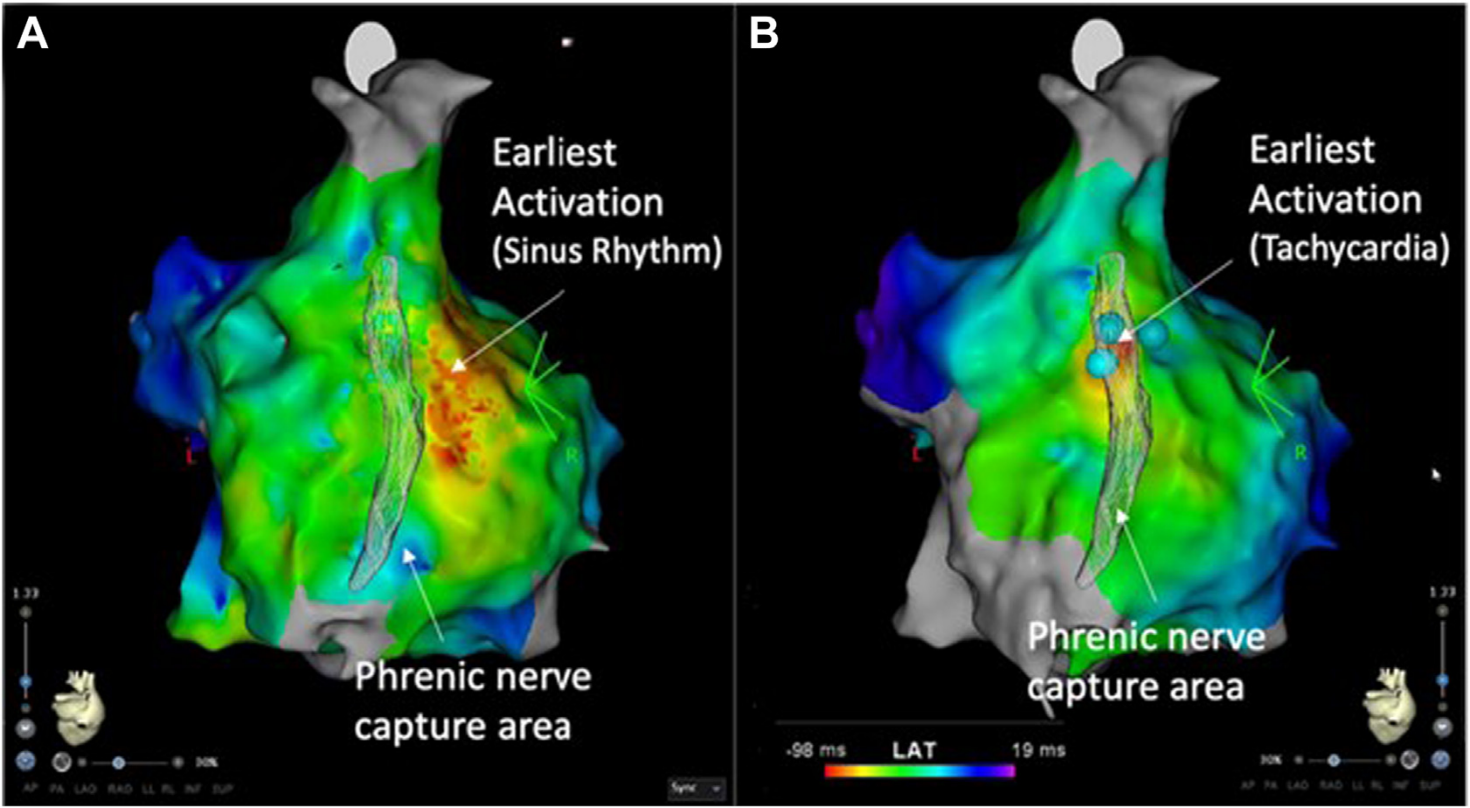
Three-dimensional electroanatomical map (CARTO; Biosense Webster) showing the proximity of focal point-by-point pulsed field ablation applications (light blue points) and phrenic nerve path (gray shadowed structure along the right atrium map) in sinus rhythm (**A**) and during atrial tachycardia (**B**).

An electrophysiology procedure was performed 1 month later with minimal sedation. After transvenous femoral access, a decapolar catheter was placed in the coronary sinus, and a quadripolar catheter in the His position. During the atrial pacing protocol (S1-S2 stimulation and short-coupled double atrial pacing), a regular supraventricular tachycardia (cycle length 290–390 ms, with 1:1 atrioventricular relationship) was induced, with concentric activation on the coronary sinus catheter and long ventriculoatrial timing. Ventricular overdrive pacing showed repeated ventriculoatrial dissociation and a high-density mapping catheter (PentaRay catheter; Biosense Webster) was used to perform electroanatomical mapping of the right atrium (CARTO; Biosense Webster). The activation map demonstrated earliest activation on the upper side of the crista terminalis, 1 cm posterior to the sinus node. Electrical high-output stimulation in this region of early activation demonstrated extensive phrenic capture and as a result, no radiofrequency ablation was performed. We then mapped the path of the phrenic nerve using high-output stimulation with the ablation catheter (ThermoCool SmartTouch Catheter; Biosense Webster) on the lateral wall of the right atrium (Figure 3). Phrenic nerve capture areas were assessed using phrenic contraction (manual palpation and fluoroscopic diaphragmatic excursion). We applied 3 lesions of F-PFA using a current of 22 A in this region, with cessation of atrial tachycardia, which was noninducible in the aftermath of the ablation. Phrenic stimulation at the end of the procedure still demonstrated phrenic capture. The patient had no recurrence in the 6 months following the procedure.

## Discussion

In this case series, we report on 3 cases in which patients were deemed unsuitable for thermal radiofrequency ablation and cryoablation because of high risk of collateral damage owing to challenging anatomies.

In the first case, the close proximity of an aortic aneurysm with the ablation target area represented a high-risk situation. The effect of ablation of the posterior wall of the LA by different energy sources on the descending aorta has been studied by Cochet and colleagues.^8^ In MRI scans 3 hours and 3 months after ablation, the damage of the descending aorta tended to be less with PFA than with thermal energies at 3 hours and had disappeared completely at the control MRI 3 months later.^7^ Of note, this study had been performed in patients with healthy aortas. In our patient with a preexisting significant aortic aneurysm, no complications occurred after F-PFA directly above the aortic aneurysm in the direct aftermath of the procedure and in the 6 months following ablation.

In the second case, a highly symptomatic patient with AF presented with a significant gastric hernia touching the posterior part of the LA and partially occluding the left inferior PV. Thermal damage of the digestive tract is among the most feared complications and occurs mostly with the use of radiofrequency energy rather than cryoenergy. The large international POTTER-AF survey^9^ (full cohort included 1758 patients) showed that esophageal fistulas after catheter ablation of AF are rare but are associated with a high mortality without surgical or endoscopic intervention. In ex vivo experiments, human cardiomyocytes were more susceptible to irreversible electroporation by pulsed electric field than human esophageal cells.^10^ Also, in the multinational MANIFEST-PF survey^11^ (cohort of 1758 patients) and the EU-PORIA registry^12^ (cohort of 1233 patients), no esophageal complications were reported. Generally, a large gastric hernia is considered as a contraindication for most centers performing AF ablations and those patients have been excluded from studies and registries on PFA ablation. Although we did not perform endoscopy to assess the presence of secondary lesions in our case, the patient did not suffer digestive complications, and the MRI performed at 3 months confirmed the absence of secondary lesions.

In the third patient, ablation of a right atrial tachycardia at the crista terminalis by thermal energy was deemed too risky because of the close proximity between the spot of earliest activation and the site of maximal phrenic nerve capture during endocardial high-output pacing. In the case of point-by-point radiofrequency ablation, the risk of phrenic nerve injury is particularly high when intra-atrial electrical high-output stimulation is associated with phrenic capture. In this case, more invasive strategies for epicardial protection of the phrenic nerve, such as manual phrenic nerve replacement or the use of balloons in the pericardial space, are used to allow safe endocardial ablation of crista terminalis atrial tachycardias. Early human studies using PFA for AF ablation found no secondary phrenic damage.^13^ Also, in the multinational MANIFEST-PF survey and the EU-PORIA registry, only 4 phrenic nerve injuries persisted post hospital discharge. All these data are derived from patients undergoing PVI with single-shot PFA devices. Despite 1 case report describing successful and safe endocardial PFA energy delivery directly at the site of maximal phrenic nerve capture,^14^ safety data remain poor in this situation. In our patient, phrenic stimulation at the end of the procedure still demonstrated phrenic capture (manual diaphragmatic palpation and diaphragmatic excursion on fluoroscopy) without any signs of phrenic nerve injury during follow-up.

All these anatomical particularities are typically exclusion criteria for thermal ablation in larger prospective studies and registries. Therefore, data on the safety of ablation procedures in these clinical settings are sparse and evidence is and will be mainly limited to case series like ours. Most studies of PFA have been performed in patients undergoing PVI with single-shot catheters (Farapulse; Boston Scientific or Affera; Medtronic). In our case series, we used a conventional 4-mm-tip ablation catheter, through which we delivered F-PFA energy using the focal PFA generator (CENTAURI; Galvanize EP, San Carlos, CA). Initial experience with this system is promising,^15^ with steep learning curves and good AF rhythm outcomes. F-PFA ablation allows the creation of lesion sets tailored to the challenging anatomies in our case series, which positions F-PFA as a useful and important tool to manage patients who may not be treatable with thermal radiofrequency ablation and cryoablation strategies.

## Conclusion

This case series adds information on the feasibility and safety profile of PFA as an energy source for focal point-by-point catheter ablation in patients with challenging anatomies, which are typically not represented in ablation studies and registries and which are often deemed unsuitable for thermal radiofrequency ablation and cryoablation because of high risk of collateral damage.

## Disclosures

The authors have no conflicts to disclose.
